# Grow or go? Energetic constraints on shark pup dispersal from pupping areas

**DOI:** 10.1093/conphys/coab017

**Published:** 2021-04-28

**Authors:** M N McMillan, J M Semmens, C Huveneers, D W Sims, K M Stehfest, B M Gillanders

**Affiliations:** 1 Southern Seas Ecology Laboratories, School of Biological Sciences, & Environment Institute, University of Adelaide, Adelaide 5005, Australia; 2 Queensland Department of Agriculture and Fisheries, Animal Science, EcoSciences Precinct, Dutton Park 4102, Australia; 3 Fisheries and Aquaculture Centre, Institute for Marine and Antarctic Studies, University of Tasmania, Taroona 7053, Australia; 4 Southern Shark Ecology Group, College of Science and Engineering, Flinders University, Bedford Park 5042, Australia; 5 Marine Biological Association of the United Kingdom, The Laboratory, Citadel Hill, Plymouth PL1 2PB, UK; 6 Ocean and Earth Science, University of Southampton, National Oceanography Centre Southampton, Waterfront Campus, Southampton SO14 3ZH, UK

**Keywords:** Connectivity, cost of transport, marine-protected areas, metabolic rate, nursery areas, ontogenetic habitat shift

## Abstract

Many sharks and other marine taxa use natal areas to maximize survival of young, meaning such areas are often attributed conservation value. The use of natal areas is often linked to predator avoidance or food resources. However, energetic constraints that may influence dispersal of young and their use of natal areas are poorly understood. We combined swim-tunnel respirometry, calorimetry, lipid class analysis and a bioenergetics model to investigate how energy demands influence dispersal of young in a globally distributed shark. The school shark (a.k.a. soupfin, tope), *Galeorhinus galeus*, is Critically Endangered due to overfishing and is one of many sharks that use protected natal areas in Australia. Energy storage in neonate pups was limited by small livers, low overall lipid content and low levels of energy storage lipids (e.g. triacylglycerols) relative to adults, with energy stores sufficient to sustain routine demands for 1.3–4 days (mean ± SD: 2.4 ± 0.8 days). High levels of growth-associated structural lipids (e.g. phospholipids) and high energetic cost of growth suggested large investment in growth during residency in natal areas. Rapid growth (~40% in length) between birth in summer and dispersal in late autumn–winter likely increased survival by reducing predation and improving foraging ability. Delaying dispersal may allow prioritization of growth and may also provide energy savings through improved swimming efficiency and cooler ambient temperatures (daily ration was predicted to fall by around a third in winter). Neonate school sharks are therefore ill-equipped for large-scale dispersal and neonates recorded in the northwest of their Australian distribution are likely born locally, not at known south-eastern pupping areas. This suggests the existence of previously unrecorded school shark pupping areas. Integrated bioenergetic approaches as applied here may help to understand dispersal from natal areas in other taxa, such as teleost fishes, elasmobranchs and invertebrates.

## Introduction

Natal areas play important roles in the life histories of many marine taxa by providing food, shelter, and protection from predation to maximize recruitment of young into adult populations (Beck *et al*., 2001; Heithaus, 2007; Nagelkerken *et al*., 2015). Recruitment from natal areas can aid recovery of depleted marine populations, and as a result they are increasingly protected as habitats of conservation importance (Garla *et al*., 2006; McLeod *et al*., 2009; White, 2015). Understanding drivers behind the use of natal areas can therefore provide valuable insights to conservation planning and management. Natal areas are often characterized by little or no overlap between young and older age classes that may present intraspecific competition or predation risks (Dahlgren *et al*., 2006; Speed *et al*., 2010; Guttridge *et al*., 2012). In these cases, recruitment of young into the broader population is dependent on dispersal from natal areas into habitats used by older conspecifics (Simpfendorfer and Milward, 1993; Eggleston, 1995; Gillanders *et al*., 2003). Such ontogenetic habitat shifts can entail substantial movements, requiring energy-intensive dispersal to forge connectivity between natal and other areas.

Because dispersal of young may be costly, it may be limited by energetic constraints that influence the use of natal areas. In sharks, the liver is the primary organ of energy storage (Sargent *et al*., 1973; Zammit and Newsholme, 1979). Individuals with large livers rich in energy storage lipids are considered in good condition and best prepared to undertake dispersive movements (Rossouw, 1987; Hoffmayer *et al*., 2006). Variation in the effects of season and location on metabolic demands, e.g. due to varying water temperature and other factors that assist or hinder dispersal such as ocean currents, may play important roles in the cost and timing of dispersal. Ecological characteristics and lifestyles of shark species can also influence energy flow between shark populations and their communities, e.g. pelagic and migratory species are likely to require more energy to fuel more active lifestyles and wide-ranging movements than less-mobile species (Cortés and Gruber, 1990; Killen *et al*., 2010).

**Figure 1:**
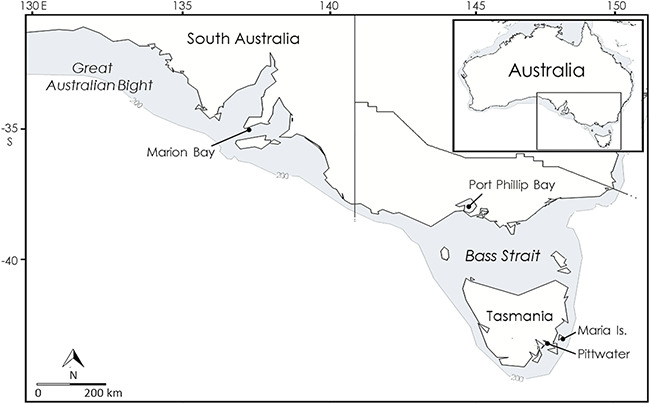
The core range of the school shark in Australia. Marked are the pupping areas in Pittwater estuary where this study was conducted; the Maria Island monitoring station on the dispersal route: Port Phillip Bay, the most westerly recorded pupping area; and Marion Bay, the location where neonate school sharks have been recorded off South Australia. The continental shelf is shaded. Inset shows the study area (boxed) relative to Australia.

The school shark (*Galeorhinus galeus*) is distributed circumglobally including in Australian waters where they undertake large-scale movements, extending from the Great Australian Bight to New Zealand (Olsen, 1954; Walker, 1999; McMillan *et al*., 2019). Their bentho-pelagic behaviour utilizes the entire water column from the sea floor to the surface with adults moving between these habitats throughout the diel cycle to forage. Pupping occurs in austral summer in sheltered bays and estuaries in the southeast of the species’ Australian range. From these pupping areas around Tasmania and Bass Strait neonates disperse, eventually mixing throughout their Australian distribution (Olsen, 1954; Stevens and West, 1997) (Fig. 1). Juvenile teleost fishes associated with inshore flats and benthic vegetation, e.g. whiting (Sillaginidae) and flounder (Pleuronectidae), are important prey for neonates departing pupping areas (Stevens and West, 1997). On this basis, it is assumed that dispersing neonates move along the coastal shelf in the relatively shallow photo-benthic zone. While most neonates depart pupping areas in autumn and winter, up to a third may return to adjacent areas as juveniles the following spring suggesting limited movements, but dispersive individuals move further (McAllister *et al*., 2015). Numerous pupping areas have been identified and protected as shark refuge areas designed and managed by the Tasmanian Department of Primary Industries, Parks, Water and Environment to protect critical reproductive habitats and maximize survival of neonates and pregnant female sharks (DPIPWE, 2020).

Because school sharks move large distances and exploit the entire water column, they are exposed to anthropogenic threats over large areas and a wide range of depths. However, their potential to recover from population depletion is limited by biological traits shared with many sharks including slow growth, late maturity and low reproductive capacity. As a result, the school shark is Critically Endangered (IUCN, 2020) with evidence of overfishing throughout its range including in California (Walker, 1998), Great Britain (Molfese *et al*., 2014) and Argentina (Cuevas *et al*., 2014). In Australia, the school shark has not recovered from population collapse in the 1990s despite cessation of targeted commercial fishing since 2001, introduction of a national recovery plan in 2008 and receiving Conservation Dependent status in 2009 (Huveneers *et al*., 2013; McAllister *et al*., 2018). Recent records of neonates in the Great Australian Bight in the northwest of their range raises several questions: (i) Does long-distance dispersal occur immediately post-birth? (ii) Are there previously unknown and unprotected pupping areas in the Great Australian Bight? (Fig. 1; McMillan *et al*., 2018). We used school sharks as a model species to investigate constraints on shark pup dispersal from pupping areas.

We conducted bioenergetic analyses on neonate school sharks from their most productive recorded pupping area in south-eastern Australia, Pittwater estuary in Tasmania (Stevens and West, 1997) (Fig. 1), to investigate constraints on dispersal from pupping areas. We hypothesized that residency in pupping areas may be influenced by energetic constraints in neonate sharks that leave them ill-equipped to disperse long distances following birth, thereby delaying dispersal. We used swim-tunnel respirometry to examine costs of transport, optimal swimming speed and routine energetic costs and conducted bomb calorimetry and lipid class analysis to assess energy storage. Finally, we calculated an energy budget for neonate school sharks to gain insight into their energetic requirements and related foraging demands and to assess how environmental conditions may influence dispersal. To our knowledge, this is the first study using such a combined energetics approach to investigate post-natal dispersal, providing potential to complement tracking studies (e.g. McAllister *et al*., 2015) by improving knowledge of drivers behind dispersal from natal areas.

## Materials and methods

### Sample collection

We used baited longlines to catch neonate school sharks in upper Pittwater estuary, Tasmania, over a 3-week period in early austral autumn (15 March–7 April 2017). The estuary has an area of 20.7 km^2^ and is characterized by shallow flats (depth, ~ 4 m) draining at low tide into a main channel (depth, ~ 8 m) (McAllister *et al*., 2018). We transported 10 neonates live to the Institute for Marine and Antarctic Studies facility at Taroona, Hobart, for respirometry trials. For bomb calorimetry and lipid analyses combined, we euthanized 13 further neonates. We then recorded sharks’ total weight (M_T_), total length, sex, liver whole wet weight (M_L_) and hepato-somatic index (M_L_/M_T_). After desiccating liver sub-samples in a freeze dryer for 5 days, we then homogenized them and stored them frozen in sealed vials at −20°C.

### Cost of transport

It is necessary to estimate routine metabolic costs to calculate an energy budget and assess relative investment of resources in functions such as energy storage and growth (Dowd *et al*., 2006). Where swimming speeds in the wild are unknown, respiration rates at optimal swimming speeds, at which cost of transport (COT) is minimal, may be used to estimate routine energy consumption (Videler and Nolet, 1990; O’Dor, 2002; Ikeda, 2016). We therefore used swim-tunnel respirometry to estimate routine energy consumption for neonate sharks. We housed sharks (*n* = 10, mean ± SD: 42.8 ± 2.2 cm total length, 0.36 ± 0.04 kg) in a 10 000 L holding tank at environmental temperatures (16–18.6°C) and fed them jack mackerel (*Trachurus declivis*) fillets once daily. Prior to respirometry trials we acclimated sharks at a controlled temperature (mean: 19.1°C, range: 18.8–19.7°C) for 24 h during which food was withheld, sufficient to allow for gastric evacuation of fillets (Schurdak and Gruber, 1989) and ensure sharks were in a post-absorptive state during trials.

We conducted trials in a 175 L, sealed recirculating Brett-type swim-tunnel respirometer with an 875 x 250 x 250 mm swim chamber (Loligo Systems, Denmark). During trials, we measured dissolved oxygen using a Witrox oxygen meter, with an optical fibre oxygen sensor (Loligo Systems, Denmark) and recorded it throughout to determine oxygen consumption rate. We flushed and refreshed respirometer water whenever oxygen saturation levels fell below 80% (as per Clark *et al*., 2013) and completed blank runs for 12 hours prior to each swim trial to assess background respiration. We introduced sharks into the respirometry chamber and acclimated them at low speeds of 0.3–0.4 body lengths per second (bl s^−1^) for 30–47 minutes until oxygen consumption reached a steady state (Johansen and Jones, 2011) before starting swimming trials. To minimize disturbance, we ran trials behind black curtains under constant red-light conditions with water temperature maintained at 20°C. Starting at 0.5 bl s^−1^, we increased swimming speed in increments of 0.1 bl s^−1^ and swam sharks at each speed for 15 minutes (as per Payne *et al*., 2011) unless the trial was terminated. Trials were terminated when sharks became exhausted, as indicated by sharks being close to the rear surface of the swim chamber for >20 seconds (Lee *et al*., 2003) or swimming in bursts (Bouyoucos *et al*., 2017), suggesting an anaerobic response (Skomal and Bernal, 2010).

To account for the increased water speed caused by the profile of the animal in the respirometry chamber, we applied a solid blocking correction as per Bell and Terhune (1970): *U*_F_ = *U*_T_(1 + ε_s_), where *U*_F_ is the speed of the corrected flow and *U*_T_ is flow speed in the swim chamber without an animal. We calculated fractional error caused by solid blocking (ε_s_) as ε_s_ = 0.8λ(*A*_O_/*A*_T_)^0.5^, where λ is a constant for animal shape (= 0.5*body length/body thickness), *A*_O_ is maximum cross-sectional area of the animal and *A*_T_ is the cross-sectional area of the swim chamber (Bell and Terhune, 1970; Payne *et al*., 2011). For each 15-minute speed trial, we fitted a linear regression to the decrease in respirometer oxygen, retaining only trials where linear regressions yielded R^2^ values >0.8 for analysis. Regressions with low R^2^ values indicate non-linear declines in respirometer oxygen, e.g. due to inconsistent activity levels during trials (Svendsen *et al*., 2016). We calculated mass-specific metabolic rates using the equation

$$ {MO}_2=\frac{\left[\left({V}_r-{V}_s\right)\times \Delta{C}_{wO2}\right]\bullet \Delta{t}^{-1}}{M^{0.86}} $$

where ${MO}_2$ is metabolic rate; ${V}_r$ and ${V}_s$ are respirometer and shark volumes, respectively; $\Delta t$ is the change in time (*t*) during trials; $\Delta{C}_{wO2}$ is the change in respirometer oxygen concentration during trials; and $M$ is shark mass scaled using an exponent of 0.86 that applies to a range of shark species (Sims, 2000). We divided resulting metabolic rates (mg O_2_ kg^−1^ hr^−1^) by swim speed in km hr^−1^ to derive COT. We then fitted a second-order polynomial to the relationship between COT and swim speed (m s^−1^) for all experimental animals combined and determined the minimum of the function to obtain the optimal swim speed at which COT was lowest (*U*_opt_).

### Calorimetry and lipid class analyses

We determined liver energy content of sharks (*n* = 13, mean ± SD: 43.3 ± 2.6 cm total length, 0.33 ± 0.05 kg) and caloric tissue value using a semi-micro oxygen bomb calorimeter (Parr model 6725, Parr Instrument Company, IL, USA) coupled with a calorimetric thermometer (Parr model 6772). We pressed sub-samples of dried and homogenized liver (~40 mg) into pellets with a 200-mg spike of known energy content to act as a fuse (standardized benzoic acid, Parr Instrument Company, IL, USA) and combusted pellets in the bomb calorimeter to yield measures of gross heat (MJ kg^−1^). By subtracting the known heat production from fuse material, we calculated liver sample energy. To calibrate the calorimeter, we combusted a benzoic acid pellet of known energy content prior to each session. We derived dried liver mass (D_L_) using the equation D_L_ = D_S_M_S_^−1^M_L_, where D_S_ was dried sub-sample mass (g), M_S_ was wet sub-sample mass (g) and M_L_ was wet liver mass (g) (Hoffmayer *et al*., 2006). We then calculated liver energy storage (E_L_) from E_L_ = D_L_E_S_, where E_S_ was dried sub-sample energy. To assess drivers of energy storage, we used a linear model with terms: E_L_ ~ length + hepato-somatic index + lipid content (i.e. percentage of liver tissue composed of lipid). Weight was highly correlated with length (*r* = 0.85), so we omitted weight as a predictor of energy storage in the model. To determine energy invested in growth, we obtained the caloric tissue value for neonates by applying the above calorimetry methodology to 40 mg sub-samples from 3 neonates homogenized whole (3 sub-samples per neonate).

We extracted lipids from sub-samples of dried and homogenized liver tissue (~0.1 g) using a modified Bligh and Dyer (1959) technique. We added sub-samples to a solvent mixture of 9 ml purified H_2_O and 20 mL methanol in valve-sealed glass funnels then agitated them gently and left them to stand for 1 h before adding 10 mL dichloromethane (DCM), agitating gently and allowing to stand overnight. After shaking funnel contents, we added 10 mL DCM and 9 mL saline purified H_2_O and left funnels to stand for 2 h. Using a rotary evaporator, we drained and concentrated contents before adding 2 mL DCM and pipetting the contents into pre-weighed sealed vials. We then expelled moisture using N_2_ flow and weighed total lipid extract prior to adding 0.5 mL DCM and storing in a freezer. To analyse lipid classes (hydrocarbons/wax esters/sterol esters, triacylglycerols, free sterols, di/monoacylglycerols and phospholipids) we used an Iatroscan Mk V TLC-flame ionization detector (Iatron Laboratories, Tokyo) after spotting total lipid on silica rods and developing solvents. We calibrated the detector using a standard mixture containing lipid classes. We then quantified lipid classes using the Iatroscan integrating software v7.0 (Iatron Laboratories, Tokyo).

### Energy budget

We calculated an energy budget by adapting the formula from Lowe (2002), including specific dynamic action, i.e. energetic costs associated with digestion: C = M + Ms + W + G, where C (energy consumed) is equal to the sum of energy used in metabolism (*M*), specific dynamic action (*M*_s_), energy lost as waste (*W*), and energy invested in growth (*G*). Because fish routinely swim at optimal speeds where energetic costs are minimal (*U*_opt_) (Videler, 1993; Clark and Seymour, 2006), COT at *U*_opt_ provides an ecologically relevant measure of energy demands in the natural environment (Steffensen, 2005). We therefore derived routine metabolic energy consumption (*M*) from COT at *U*_opt_ (COT at *U*_opt_ * *U*_opt_), as a proxy for routine metabolic rate (Ikeda, 2016) and scaled metabolic rate to mean animal size (g) using a mass scaling exponent of 0.86 (Sims, 2000).

Neonates have been observed to disperse from Pittwater north along the coastal shelf as evidenced by acoustic detections at the Maria Island monitoring station (McAllister *et al*., 2015) (Fig. 1). To predict effects of spatial and seasonal changes in temperature we applied a temperature coefficient (Q_10_) of 2.51 derived from resting data (as per Dowd *et al*., 2006) from the closely related leopard shark (*Triakis semifasciata*)*,* that inhabits a similar thermal range (Miklos *et al*., 2003). Elasmobranch metabolic rates generally increase by a Q_10_ in the range of 2–3 (Carlson *et al*. 2004). We made adjustments for mean water temperatures in early autumn (1 March–15 April: 17.2°C) and late autumn (16 April–31 May: 12.6°C) in Pittwater (Semmens, unpublished data) and in early autumn (17.4°C), late autumn (15.3°C) and winter (1 June–15 July: 13°C) at Maria Island (depth: 20 m; IMOS, 2018) (Fig. 1), representing conditions on the dispersal route. We also modelled adjustments for current strength and direction on the dispersal route where the East Australia Current flows in a mean poleward direction at Maria Island during autumn–winter by approximating incoming flow to reduce ground speed by a corresponding amount (at 20 m depth, mean direction: 161°, mean flow: 0.21 m s^−1^; IMOS, 2018).

We calculated specific dynamic action costs for neonates at 6% of metabolic energy consumption (Sims and Davies, 1994) and energy lost to waste at 28% including faecal and nitrogenous wastes and egestion (Wetherbee and Gruber, 1993). Growth was derived from a non-linear least squares model applied to shark lengths surveyed in Pittwater estuary from 2011 to 2017 (Fig. S1). Since male and female school shark growth curves do not differ (Moulton *et al*., 1992), we derived growth in mass from the weight–length relationship for school sharks: y = 4.86(10^−6^x^3.18^) where y = weight (lb) and x = length (cm) (Olsen, 1954). We then converted weight to grams and multiplied weight by the caloric tissue value we obtained for school sharks (5.8 kJ g^−1^). Since pups are immature, we calculated all energy devoted to growth as somatic rather than reproductive growth.

## Results

### Swimming performance and energy budget

**Figure 2:**
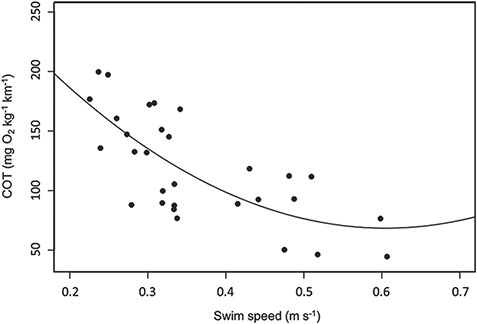
COT (mg O2 kg wet weight^-1^ km^-1^) as a function of swimming speed (m s^-1^) for neonate school sharks from swim-tunnel respirometry trials; a polynomial trendline was fitted to derive optimal swimming speed at which COT was lowest (Uopt).

At 20°C, *U*_opt_ was 0.6 m s^−1^ (Fig. 2), equating to a mean of 1.4 bl s^−1^ and metabolic rate at *U*_opt_ was 149 mg O_2_ kg^−1^ h^−1^. Adjusting for seasonal differences in ambient water temperature on the coastal dispersal route yielded predicted decreases in metabolic rate at *U*_opt_ that ranged from 122 mg O_2_ kg^−1^ h^−1^ in early autumn (17.4°C) to 78 mg O_2_ kg^−1^ h^−1^ in winter (13°C). COT at *U*_opt_ on the coastal dispersal route decreased from 0.7 J g^−1^ km^−1^ in early autumn to 0.5 J g^−1^ km^−1^ in winter. Adjustment for swimming into the poleward flowing East Australia Current on the dispersal route (mean flow rate: 0.21 m s^−1^) gave a COT of 0.9 J g^−1^ km^−1^ in early autumn decreasing to 0.6 J g^−1^ km^−1^ in winter.

Growth ranged from 2.64 to 2.99 g day^−1^ and was the largest energetic cost, demanding 15.3–17.3 kJ day^−1^ (Table 1). Metabolic energy consumption ranged from 10.7 to 15.3 kJ day^−1^ and energy lost to waste ranged from 10.6 to 12.7 kJ day^−1^ yielding a total routine energy consumption of 38–45.4 kJ day^−1^ (Table 1). Whiting, the most important prey item for dispersing neonate school sharks (Stevens and West, 1997), had a mean caloric value of 5.9 kJ g^−1^ (McCluskey *et al*., 2016). Based on this, neonates would need to consume 6.4–7.7 g prey day^−1^ to satisfy routine energy requirements, i.e. a daily ration of 1.5%–2.3% wet bodyweight (Table 2). Since mean-sized whiting prey for neonate school sharks is 13 g (Semmens, unpublished data), this would require a successful hunt approximately every 2 days.

**Table 1:** Modelled energetic parameters for neonate school sharks of mean size in Pittwater estuary in early autumn (1 March–15 April) with adjustments for changes in site (Maria Island on the dispersal route) and season (late autumn: 16 April–31 May; winter: 1 June–15 July)

Site	Season	Temp, °C	Length, cm	Weight, g	Metabolism, kJ day^−1^	SDA, kJ day^−1^	Growth, kJ day^−1^	Waste, kJ day^−1^	Total, kJ day^−1^	Daily ration % wbw
Pittwater	Early autumn	17.2	43.3	330	15.1	0.9	15.3	12.2	43.5	2.2
	Late autumn	12.6	46.3	436	10.7	0.6	15.8	10.6	37.8	1.5
Maria Is.	Early autumn	17.4	43.3	330	15.3	0.9	15.4	12.3	43.9	2.3
	Late autumn	15.3	46.3	436	14.7	0.9	15.6	12.2	43.3	1.7
	Winter	13.0	48.7	512	14.3	0.9	17.3	12.7	45.1	1.5

**Table 2:** Liver energy and lipid stores of neonate school sharks from Pittwater estuary including total length, weight, sex (male/female), liver wet weight, hepato-somatic index (HSI), lipid content (percentage of liver tissue composed of lipid), total stored energy (total energy stored in livers of each shark) and number of days energy stores are calculated to last without further feeding when sampled in Pittwater in early autumn. Bottom row provides means ± SD for all parameters except sex, where M:F ratio is provided

Length, cm	Weight, g	Sex, M/F	Liver wet wt., g	HSI, %	Lipid content, %	Total stored energy, kJ	Energy stores, days
42	323	M	9.1	2.8	34.7	67	1.7
42	325	M	7.7	2.4	29.7	62	1.5
46	384	F	18.3	4.8	44.7	247	4.0
41	333	M	10.4	3.1	26.1	86	2.8
47	386	M	13.2	3.4	56.2	175	2.5
41	306	F	11.2	3.7	33.9	91	2.5
44	363	F	12.3	3.4	35.6	133	2.8
40	253	F	11.0	4.4	44.7	128	3.1
39	228	F	7.7	3.4	37.5	69	1.7
46	401	M	18.9	4.7	52.4	187	3.3
45	344	M	14.7	4.3	34.1	117	1.9
46	369	F	11.7	3.2	39.6	127	2.0
44	282	M	10.1	3.6	34.1	84	1.3
43 ± 2.6	330 ± 52.6	7:5	12 ± 3.5	3.6 ± 0.7	38.7 ± 8.7	121 ± 54.8	2.4 ± 0.8

### Calorimetry and lipid class analyses

**Figure 3:**
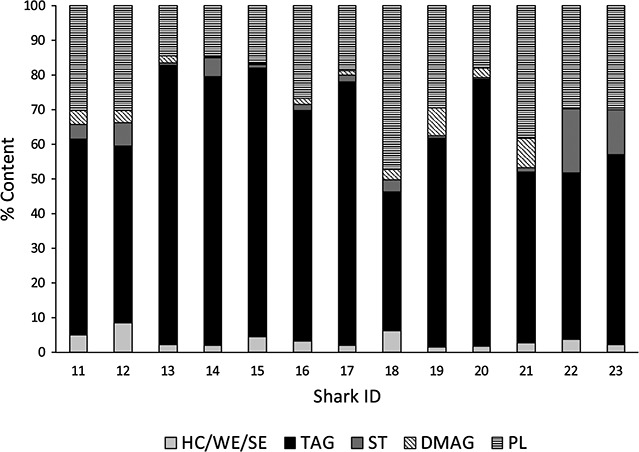
Lipid class profiles as % total liver lipid content for neonate school sharks (HC , hydrocarbons; WE, wax esters; SE, sterol esters; TAG, triacylglycerols; ST , free sterols; DMAG , di/monoacylglycerols; PL, phospholipids; identification numbers for sharks are given on the x-axis)

Livers of neonates were small with a mean hepato-somatic index of 3.6% wet bodyweight (range, 2.4%–4.8%). Mean stored liver energy was 120.9 ± 54.8 kJ (range, 59.8–249.8 kJ) (Table 2). The linear model using lipid content, hepato-somatic index and length as explanatory variables explained 76% of the variance in stored energy (*R*^2^ = 0.76, F_(3,9)_ = 13.56, *P* < 0.01). Energy increased by 1.6 kJ per % increase in lipid content (mean ± SD: 38.7 ± 8.6%), 35 kJ per % increase in hepato-somatic index (3.6 ± 0.7%), and 8.5 kJ per cm increase in length (43.3 ± 2.6 cm). Lipid class profiles were broadly similar with triacylglycerols and phospholipids most abundant, however, proportions varied among individuals (Fig. 3). Mean content (± SD) of lipid classes were as follows: triacylglycerols, 62.71 ± 13.9%; free sterols, 4.56 ± 5.4%; hydrocarbons/wax esters/sterol esters, 3.47 ± 2.1%; di/monoacylglycerols, 3.23 ± 2.8%; and phospholipids, 26.52 ± 9.7%. Mean energy stores at the time of sampling were sufficient to sustain routine energy requirements for 2.4 ± 0.8 days without further feeding but differed among individuals (1.3–4 days; Table 2).

## Discussion

Neonate sharks were ill-equipped for long-distance dispersal due to their low energy stores, characterized by small livers, low overall lipid content and low levels of energy storage lipids relative to adults. Substantial investment of available resources in growth (the largest energetic cost) and high levels of growth-associated structural lipids were also found. Pups grew rapidly in the pupping area, increasing length by ~40% from birth in summer through to dispersal in late autumn (Fig. S1). Delaying dispersal in neonate sharks thus appears to allow prioritization of growth. These findings are supported by the tendency of young sharks of numerous species (Kinney and Simpfendorfer, 2009) including school sharks (Thorburn *et al*., 2019) to maintain limited home ranges. Delaying dispersal and prioritizing growth likely increases survival, since growth offers advantages for foraging and intra-specific competition while reducing predation risks at this vulnerable life stage (Morrissey and Gruber, 1993; Heupel *et al*., 2007). Delaying dispersal may also confer energetic benefits when dispersal eventually occurs. In addition to allowing time to build energy stores to sustain long-distance travel, swimming costs also decrease with increasing mass (Schmidt-Nielsen, 1984). Furthermore, cooling ambient water temperatures were predicted to reduce routine energy costs, reducing daily ration requirements by around a third from early autumn to winter (Table 1). Low lipid stores also indicate low buoyancy, which is reflected in the benthic lifestyle of neonate school sharks and not conducive to efficient swimming, further indicating the ill-preparedness of neonates to disperse long distances.

The liver is the main site of energy storage in elasmobranchs, where lipids are synthesized and stored to fuel metabolic activity (Sargent *et al*., 1972; Zammit and Newsholme, 1979). As such, shark livers are particularly energy rich, e.g. white shark (*Carcharodon carcharias*) livers have higher energy density than whale blubber (Pethybridge *et al*., 2014). Liver energy stores (and thus liver size) are depleted to fuel energy-intensive tasks including dispersal (Bone and Roberts, 1969; Rossouw, 1987, Del Raye *et al*. 2013), meaning that mature school sharks have significantly smaller livers after migrating (Olsen, 1954). Liver lipids are also used to offset starvation with individuals in poor condition having small livers (Bone and Roberts, 1969; Hoffmayer *et al*., 2006). Neonate livers were 3–6 times smaller than adult livers relative to body mass (adult hepato-somatic index, 10%–20%; Ripley, 1946a) and had low lipid content (~39% in neonates *v*. ~60% and ~75% in adult males and females, respectively; Ripley, 1946b). In addition to low energy stores, low lipid levels indicate high body density and low hydrostatic lift, suggesting a predominantly benthic lifestyle (Bone and Roberts, 1969; Rossouw, 1987). Larger livers increase static buoyancy (lift), reducing dynamic lift costs of more active swimming and increasing swimming efficiency (Iosilevskii and Papastamatiou, 2016). Increased buoyancy facilitates exploitation of the water column as seen in the ubiquitous diel vertical foraging of adult school sharks (McMillan *et al*., 2019). Conversely, lower buoyancy in neonates is reflected in their diet comprising mainly benthic taxa (Stevens and West, 1997; McAllister *et al*., 2015) and may also assist predator avoidance by maintaining position near the seafloor. Small livers and low lipid stores therefore appear to be key constraints on dispersal by limiting energy stores and swimming efficiency.

**Figure 4:**
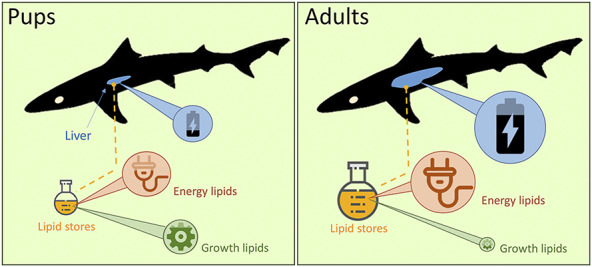
Energy storage in shark pups (left) was limited by small livers relative to body size, low lipid stores relative to liver size, low levels of energy storage lipids and high levels of growth-associated lipids compared to adults; these constraints appear to limit dispersal from pupping areas while growth is prioritized, paying off through increased survival, improved swimming efficiency, and lower costs of later dispersal.

High proportions of structural lipids *v*. energy storage lipids in neonates relative to adults further supports prioritization of growth over energy storage (Fig. 4). While energy storing triacylglycerols were in greatest abundance, comprising nearly two thirds of liver lipids, this was far lower than in adult school sharks where they comprise >95% of liver lipids (Nichols *et al*., 1998). Conversely, structural phospholipids that are important components of cell membranes and thus growth (Pethybridge *et al*., 2010) were the second most abundant lipids in neonates at ~26% compared to just 2% in adults (Nichols *et al*., 1998). Crustaceans and cephalopods were roughly of equal importance to small teleost fish in the diet of neonate school sharks in Pittwater (Stevens and West, 1997), but yield low lipid content compared to teleost prey and cephalopod flesh in particular yields mainly structural lipids (Semmens, 1998). Teleost fish become increasingly important in the diet of juveniles as they grow (Stevens and West, 1997), marking a transition from generalist foraging in inexperienced neonates to a more specialized focus on higher energy teleost prey as foraging ability increases. The high levels of structural lipids found in this study confirm that this transition is yet to occur in neonates in the pupping area and further support a low preparedness for energy-intensive long-distance dispersal.

In addition to increasing survival and foraging ability, delaying dispersal to grow offers other benefits for subsequent dispersal in terms of increased swimming efficiency. As fish increase in size, their surface-to-volume ratio decreases, contributing to a lower COT. In sharks, this can be approximated by an exponent of ~ 0.3 (Schmidt-Nielsen, 1984). Seasonal and spatial changes in ambient water temperature can also have strong effects on energy consumption in ectothermic sharks (Carlson and Parsons, 1999; Miklos *et al*., 2003; Bethea *et al*., 2007). Our bioenergetics model predicted considerable energetic savings by delaying dispersal until cooler ambient temperatures occurred on the dispersal route in late autumn and winter, consistent with thermal effects of decreasing water temperature lowering metabolic rate and swimming costs (Clark and Seymour, 2006). These predicted energetic savings may be conservative, because although our model assumed constant swimming speed, swimming efficiency may increase at cooler temperatures (Dickson *et al*., 2002; Clark and Seymour, 2006). Ration levels may also increase at lower latitudes due to increasing ambient temperatures elevating metabolic demands (Bethea *et al*., 2007). Increasing energetic costs for neonates as they move north along the Tasmanian coast into warmer waters may therefore provide further reason to delay dispersal until temperatures fall.

The optimal swimming speed of 1.4 bl s^−1^ was comparable to other ectothermic sharks of similar size (0.9–1.7 bl s^−1^) including scalloped hammerheads (*Sphyrna lewini*) (Lowe, 1996), lemon sharks (*Negaprion brevirostris*) and leopard sharks *T. semifasciata* (Graham *et al*., 1990). Optimal swimming speed of neonates was near the maximum sustained swimming speed recorded, suggesting neonate swimming performance does not allow energetically optimal swim speeds substantially lower than maximal sustainable cruising speeds. It may be that the low buoyancy (small livers with low lipid content) and/or limited hydrodynamic performance (small, floppy pectoral fins, underdeveloped and not conducive to maintaining position in the water column) affect the swimming performance of neonates and push their optimal swimming speed up towards their maximal performance. Whitney *et al*. (2016) similarly recorded limited sustainable swim speeds beyond optimal swimming speed in juvenile nurse sharks (*Ginglymostoma cirratum*) at 30°C. Although the optimal swimming speed for neonates suggests a fast theoretical dispersal capacity (up to ~41 km day^−1^ swimming into the East Australia Current at mean flow), such speeds are unlikely to be achieved – even migrating adult school sharks moved at a maximum speed of 24 km day^−1^ (McMillan *et al*., 2019). Acoustically tracked neonates dispersing from Pittwater covered the 155 km to Maria Island at a fastest dispersal rate of 3.5 km day^−1^ (McAllister *et al*., 2015). Sharks in the wild are not forced to maintain position in a current (as in swim-tunnel respirometers) and often exploit the vertical water column when swimming, e.g. ascending against gravity before glide descending, offering foraging and energetic benefits but slowing horizontal swimming speeds (Barnett *et al*., 2010). Carcharhiniform sharks are also capable of both ram ventilating while swimming and buccal pumping while at rest (Carrier *et al*., 2012), so swimming speeds from trials cannot be easily equated to daily dispersal rates. Neonates were observed resting in holding tanks and undertake limited movement during daylight in the wild (Barnett and Semmens, 2012), suggesting continuous swimming by neonate school sharks in the wild is unlikely.

Prioritization of growth and small energy stores thus appear to constrain dispersal in shark pups until sufficient growth and energy storage occur or favourable ambient conditions, e.g. water temperature or currents, reduce energetic costs. Field observations of neonate school sharks support an incremental dispersal from pupping areas rather than direct, rapid dispersal. Neonates in Port Phillip Bay (Fig. 1) began congregating in channels in early autumn before meandering towards the open sea and dispersing from the bay by late winter (Olsen, 1954). In Pittwater, similar behaviour was observed with neonates beginning to move into lower reaches of the estuary during autumn and dispersing into adjacent coastal areas in late autumn and winter (McAllister *et al*., 2015). These movements are consistent with our findings of low energy stores in early autumn and the energetic benefits of delaying dispersal until water temperatures fall in late autumn and winter.

Neonate school sharks ~1–4 months old have recently been recorded in the Great Australian Bight off South Australia during the summer pupping season 840–1700 km from known pupping areas in Tasmania and Bass Strait (e.g. Rogers *et al*., 2017; McMillan *et al*., 2018). Rapid dispersal from distant pupping areas is unlikely for neonates in South Australia given our findings of low preparedness for dispersal and that at this time neonates in known pupping areas are yet to begin their autumn–winter movement towards the open sea (Olsen, 1954; McAllister *et al*., 2015). Neonate school sharks tagged in Bass Strait and Tasmania that dispersed to South Australia required 12–24 months (Olsen, 1954; Semmens, unpublished data), by which time they were no longer neonates but 1–2 year-old juveniles. Dispersing from the nearest known pupping area (Port Phillip Bay; Fig. 1) shortly after birth, neonates would need to swim up to ~60 km day^−1^ to arrive at locations where they have been recorded in South Australia in the observed size range. Additionally, the observed post-natal residency when growth occurs in pupping areas would be foregone and energetic costs would be elevated due to high summer temperatures. Immediate post-natal dispersal over such distances is therefore unlikely, suggesting undocumented local pupping areas in South Australia that could be valuable to conservation management.

This study suggests a trade-off, with shark pups delaying dispersal to prioritize growth. This is likely because growth increases survival through reduced predation and provides foraging advantages. Delaying dispersal also offers energetic benefits for subsequent dispersal through increased swimming efficiency and reduced energy demands. These findings suggest limited dispersal by neonate school sharks and are supported by both traditional mark-recapture and acoustic tracking studies (Olsen, 1954; McAllister *et al*., 2015). This study also indicates that neonate school sharks in South Australia were likely born locally in undocumented pupping areas rather than being migrants from distant pupping areas in south-eastern Australia. This has important management implications given the species’ overfished status in Australia, long projected recovery time (up to 66 years to reach 20% of virgin biomass: Thomson, 2012) and Critically Endangered status globally (IUCN, 2020). In addition to low extant biomass, it is likely that habitat degradation since the 1970s (draining of adjacent swamps, clearing of mangroves and die-back of seagrass beds) has severely diminished the contribution to the population from previously highly productive pupping areas (e.g. Port Phillip and Western Port Bays) that have shown little recovery (DEWR, 2008). Energetically mediated residency in pupping areas, as our findings suggest, further emphasizes the importance of conserving and restoring remaining pupping areas since neonate movements to less degraded habitats after birth seem unlikely. Such efforts may also include a need to identify and protect undocumented school shark pupping areas, e.g. in waters off South Australia.

We anticipate that the bioenergetic constraints on shark pup dispersal presented here will be useful to conservation management, providing insight into the biology and ecophysiology that influence residency in pupping areas. Knowledge of the energetic constraints underlying post-natal residency and dispersal could assist in the planning of marine-protected areas (particularly temporal protections). Future developments such as further miniaturization of pop-up archival tags or expansion of acoustic receiver networks may provide explicit information about dispersal of young from pupping areas in terms of routes, behaviour (e.g. direct movement *v*. foraging), rates of dispersal and destination that may have important ramifications both for this Critically Endangered species and other elasmobranchs. More generally, the approach presented here may be adapted to address conservation management issues in other marine taxa reliant on dispersal from natal areas, e.g. post-natal residency in protected areas and dispersal capacity of young, with implications for exposure to stressors in natal areas and during dispersal from them.

## Funding

This work was supported by the University of Adelaide, the University of Tasmania and by the Frederick James Sandoz Scholarship for Animal Research to M.N.M. M.N.M. was also supported through the provision of an Australian Government Research Training Program Scholarship. D.W.S. was supported by a Visiting Fellowship from the University of Tasmania and a Marine Biological Association Senior Research Fellowship.

## Supplementary Material

Pup_energetics_Supp_mat_FINAL_coab017
