# Supplementary material for: Grow or go? Energetic constraints on shark pup dispersal from pupping areas
Source: Conserv Physiol. 2021 Apr 28;9(1):coab017. doi: 10.1093/conphys/coab017 (PMC8084043; doi:10.1093/conphys/coab017)
Supplement: Pup_energetics_Supp_mat_FINAL_coab017 [file pup_energetics_supp_mat_final_coab017.docx]

**Supplementary material**

**Grow or go? Energetic constraints on shark pup dispersal from pupping areas**

M. N. McMillan, J. M. Semmens, C. Huveneers, D. W. Sims, K. M. Stehfest, and B. M. Gillanders


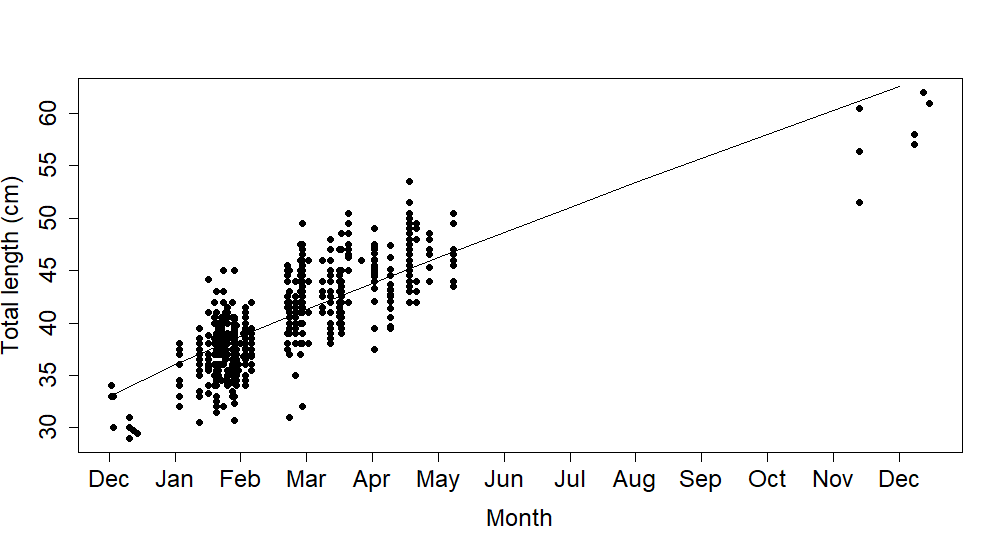


**Fig. S1.** Length of school shark pups in Upper Pittwater estuary as a function of sampling time (sampling period: 2011–2017). A non-linear least squares curve was fitted to derive growth rates for pups. Pupping occurs in austral summer (Dec–Feb). Dispersal from Upper Pittwater is completed in May. Some juveniles return to the pupping area the following spring after birth.
